# Beyond school gates: the role of motivation in music learning on elementary school students’ daily music listening behaviors

**DOI:** 10.3389/fpsyg.2025.1441572

**Published:** 2025-07-16

**Authors:** Yuki Harada, Saho Takeishi

**Affiliations:** ^1^Faculty of Education and Human Studies, Akita University, Akita, Japan; ^2^Graduate School of Education, Akita University, Akita, Japan

**Keywords:** music listening, motivation, expectancy belief, task values, music education

## Abstract

**Background:**

Research in music psychology suggests that attentive music listening cultivates deeper understanding and creativity. However, many children engage with music primarily in informal settings, so a high level of motivation for formal music learning does not necessarily translate into everyday music-listening behavior. This study examined whether motivation for formal music learning predicts students’ out-of-school music-listening behaviors, even after accounting for their motivation for informal music engagement.

**Methods:**

Participants were 1,382 elementary school students (Grades 4–6) in Japan. First, we developed a new scale to measure expectancy belief for formal music learning across five domains: instrumental performance, singing, composing, listening, and studying musical terminology. Second, we conducted a main study using structural equation modeling to test whether motivation for formal and informal music engagement (each with expectancy belief and task value) would explain two type of everyday music-listening behaviors: intention to access music and attention to musical elements.

**Results:**

Factor and correlation analysis supported the validity of the new expectancy belief scale. Structural equation modeling showed that while the intention to access music was primarily predicted by motivation for informal music engagement, the attention to musical elements was significantly explained by both informal and formal music motivation. Notably, an interaction emerged in which higher expectancy beliefs reinforced the positive effect of task value on attention to musical elements.

**Discussion:**

Although motivation for formal music learning showed little association with the intention to access music, its significant association with attention to musical elements suggests that school music education and everyday music listening are not entirely disconnected. Motivation for formal music learning may foster more analytical engagement with music in daily life, supporting broader educational goals of musical understanding and creativity.

## 1 Introduction

### 1.1 Music listening in daily life

The significance of music and the qualities of superior music have been discussed since the era of ancient Greek philosophers such as Plato and Aristotle (Stamou, 2002). It is likely that many modern individuals in the 21st century would agree that listening to music enriches human life. In fact, the sheer volume of exposure to music is arguably more significant now than at any other time in human history. For instance, in contemporary society, music is played in retail environments to encourage consumer purchases and choices (e.g., North et al., 1997; North et al., 2003), resulting in consumers’ inevitable exposure to music simply by engaging in shopping activities. Thus, the opportunities and roles of music listening in people’s daily lives are increasingly expanding and diversifying (Hargreaves and North, 1999).

Music listening is entrenched in the lives of modern individuals and youth as a form of entertainment and hobby (e.g., Brown et al., 1986; Lamont et al., 2003; North et al., 2004; Rideout et al., 2010). Tarrant et al. (2000) investigated the reasons for music listening among adolescents in America and the UK (*n* = 245, *Mean* Age = 15.27 years) and identified three factors: “self-actualization,” “fulfilling emotional needs,” and “fulfilling social needs.” Similar studies have been conducted (e.g., Greasley and Lamont, 2011; Greb et al., 2018; Ter Bogt et al., 2011), and while different purposes and functions of music listening have been occasionally revealed, the items commonly used across many surveys can be summarized into three factors akin to those reported by Schäfer et al. (2013): “self-awareness,” “arousal and mood regulation,” and “social relatedness.” These findings indicate that people spontaneously access music to fulfill their social and emotional needs as well as for self-actualization, which might not necessarily be similar to the purposes of music listening in the context of formal music education.

As noted above, previous studies have focused on the various purposes, types, and sociocultural functions of everyday music listening (e.g., Goopy and MacArthur, 2025; Hargreaves et al., 2006; Miranda, 2012; Schäfer et al., 2013). For adolescents, music listening serves as a cultural practice with multiple roles and functions (Miranda, 2012), including esthetic appreciation for music and the development of musical tastes (Hargreaves et al., 2006; Mulder et al., 2010), the formation of cultural identity and self-concept (Kistler et al., 2010), the distinction between in-groups and out-groups through musical preferences (Hargreaves et al., 2006), and emotion regulation (Garrido et al., 2022). Among these, the present study particularly focuses on the cognitive aspects of everyday music listening, namely the tendency to access and listen to music and the tendency to attend to various musical elements while listening. As discussed below, both aspects are beneficial for fostering musical knowledge and understanding. Furthermore, because listening strategies that emphasize attention to musical elements are explicitly taught in formal music education at school [in the case of Japan, Ministry of Education, Culture, Sports, Science, and Technology–Japan (MEXT), 2017], this aspect may serve as a point of intersection between students’ everyday lives and formal music education.

### 1.2 Exposure to music and attentive music listening

Research in experimental psychology and psychophysiology has demonstrated that mere exposure to music enables individuals to subconsciously learn about various musical rules (e.g., tonality: Tillmann et al., 2000; melodic expectancy through statistical learning: Kern et al., 2022) (for reviews, see Bigand and Poulin-Charronnat, 2006; Rohrmeier and Rebuschat, 2012; Thompson et al., 2023). Studies using event-related brain potentials (ERPs) have investigated the nature of music cognition-related components (e.g., Early Right Anterior Negativity, N5), indicating that even when participants do not consciously attend to music, processes related to the syntactic processing of music and harmony are automatically engaged (e.g., Koelsch, 2009, 2011). Moreover, exposure to music can enhance performance on attention and cognitive tasks, mediated by changes in the participants’ emotions and motivation (e.g., Cohen et al., 2025; Mendes et al., 2021; Schellenberg et al., 2007). Thus, the simple act of listening to music activates diverse neural processes related to music, enhances cognitive functions in a state-dependent manner, and further cultivates expertise in music appreciation. In essence, even mere exposure to music allows individuals to learn and derive benefits from it.

While exposure to music, or what might be called “just listening,” is a starting point, the step-up to “attentive music listening” is considered the foundation for the diverse creativity inherent in music (e.g., Gordon, 1989; Kratus, 2017; Peterson, 2006). Campbell (2005) stated, “Students can also come to the brink of understanding music’s formal structures, including its elements of time (rhythm) and pitch (its horizontal melodic structures and vertical chords and clusters), through attentive, engaged, and enactive listening.” In other words, attentive, analytical music listening is an indispensable act for comprehending the various elements and forms of music as well as its cultural depth, and serves as a fundamental and central learning activity in music education aimed at developing diverse competencies (e.g., knowledge, skills, thinking ability, expressiveness, and creativity) in students.

The benefits of attentive music listening extend beyond the expansion of musical abilities, offering listeners much more (Greb et al., 2018). Leipold and Loepthien (2015) investigated how two forms of music reception (attentive–analytical listening and emotional listening) are related to emotion and stress regulation. The results indicated that attentive–analytical listening has a positive association with accommodative coping, a process of adjusting goals to situational constraints, and a negative association with rumination. Another study showed that the propensity for attentive–analytical listening moderates the extent of flow experiences induced by complex music (Ruth et al., 2017).

Within the realm of music semantic processing, there are perceptual and cognitive processes that only occur when attention is directed toward the music itself. Kopiez and Platz (2009) reported that although music experts are more likely to notice a clash of keys compared to non-experts, without attention being guided to the key, even experts only detected clashes 49.3% of the time in their study (experts detected them 78.0% of the time when their attention was directed to the key). These findings indicate that conscious perception of the key, a principal element of music composition, requires the listener’s top-down attention. Koelsch et al. (2004) explored the properties of the N400, an ERP component, to ascertain whether music, like words, is capable of conveying semantic content. The N400, originally used as an index to examine language semantic processing, is a parietally centered ERP component that shows increased amplitude to target stimuli with a small semantic relation to a priming stimulus (e.g., for a review, see Kutas and Federmeier, 2011). The results showed that the N400 was elicited in response to words that were incongruent with the presumed meaning conveyed by the musical excerpts. This implies that excerpts of music, even though not explicitly “speaking,” can transmit semantics akin to language. Significantly, subsequent research found that the N400 effect, which reflects music semantic processing, is observed clearly only under conditions where participants pay attention to the semantics of the music (e.g., Daltrozzo and Schön, 2009; Painter and Koelsch, 2011). Magnetic resonance imaging (MRI) studies measuring blood-oxygen-level-dependent (BOLD) signals when manipulating music listening strategies related to attention have reported that listening while focusing on the timbre of a single instrument broadly recruits neural circuits associated with attention, working memory, semantic processing, and motor imagery (Janata et al., 2002). While caution must be exercised in directly linking findings from psychophysiology to music education, practical research findings concluding that the use of attentive and critical listening strategies has high pedagogical value in music education, as it aids in recognizing the messages (explicit or implicit) presented in music, are consistent with such insights (e.g., Beach and Bolden, 2018).

### 1.3 Music listening in music education and daily life

As previously mentioned, music bestows certain benefits even when merely listened to passively (e.g., Cohen et al., 2025), yet there are unique cognitive processes and gains that are realized only through attentive music listening. The importance of attentive music listening has been recognized in school music education; thus, students are instructed in listening strategies and methods within the curriculum (e.g., Campbell, 2005; McAnally, 2007; Svalina and Sukop, 2021; Todd and Mishra, 2013). This raises an important question: can music education in schools improve the quantity (frequency, duration, etc.) and quality (attentiveness to music, etc.) of students’ music listening in their daily lives? Considering that many students’ exposure to music occurs more often in their everyday life than in music classes (e.g., Lamont et al., 2003), this question merits attention.

To our knowledge, no study has completely answered this question thus far. However, it is known that for many students, there is a disconnect between music education in schools—formal music learning—and musical experiences in daily life—informal music engagement (e.g., Boal-Palheiros and Hargreaves, 2001; Lamont et al., 2003; Ross, 1995, 1998). It would not be surprising, albeit disappointing for music educators, if school-based music education did not have a strong influence on students’ music-listening behaviors in their daily lives. Meanwhile, music education often encourages analytical listening by directing attention to the components of music (e.g., pitch, rhythm, and timbre) (Campbell, 2005; Todd and Mishra, 2013), and sometimes utilizes music that is relatable to students’ lives (Todd and Mishra, 2013). Therefore, it would not be surprising if formal music education could influence everyday behaviors through the mediation of cultivated student motivation and attitudes.

The continuum from simple music listening to attentive music listening can be understood as a spectrum characterized by the amount of cognitive resources invested in musical stimuli. Given that previous research has focused on the various purposes, types, and socio-cultural functions of adolescents’ music listening behaviors in daily life (e.g., Goopy and MacArthur, 2025; Hargreaves et al., 2006; Miranda, 2012; Schäfer et al., 2013), this framework can be regarded as capturing only one dimension of the multifaceted nature of music listening. Nevertheless, as mentioned earlier, it is well established that even passive exposure to music can yield cognitive and affective benefits (e.g., Cohen et al., 2025), and that attention facilitates higher-order cognitive processes such as memory and semantic processing, thereby enabling additional gains from music. From this perspective, identifying predictors that promote both the intention to engage in music listening in daily life and attention to musical elements holds considerable value. In the present study, intention is treated as a concept similar to “listening-music willingness,” which has been defined in previous research as “an individual’s positive attitude and eagerness to engage in the activity of listening to music” (Wang and Huang, 2024). Importantly, intention in this context is not concerned with the qualitative aspects of music listening—such as an individual’s purpose for listening, the amount of cognitive resources invested, or other facets of cognitive processing and meaning-making. Therefore, we conceptualize intention as representing the quantitative aspect of music listening. In contrast, attention corresponds more closely to constructs such as the “rational appreciation of music” (Chamorro-Premuzic and Furnham, 2007) and “attentive–analytical listening” (Leipold and Loepthien, 2015, 2021), and reflects one of the qualitative aspects of how individuals listen to music.

If music teachers are able to enhance students’ intention or attention, school-based music education could significantly influence the way students engage with music in their daily lives. However, school education is constrained by the national curriculum, including educational goals, learning content, and instructional methods (MEXT, 2017), making it unrealistic for classroom instruction to directly target students’ “daily lives” as a domain of intervention. Therefore, the variables that music teachers can realistically influence in through their daily educational practice are psychological factors related to school-based music education. This study focuses on the motivation for school-based music education and for music listening in daily life, and examines their effect on elementary school students’ music listening behavior in their everyday lives. Resolving this question will contribute to our understanding of whether the motivational impact of school music education extends beyond the school gates to influence students’ everyday lives, or if its effects remain confined within the school context.

### 1.4 Motivation for musical activities

Academic motivation is defined as “the process whereby goal-directed [academic] activity is instigated and sustained” (Schunk et al., 2014). Not limited to music education, it is well known that student motivation robustly correlates with educationally desirable behaviors and outcomes, including academic achievement indicators and the use of cognitive and metacognitive learning strategies (e.g., Anderman and Dowson, 2011; Eccles and Wigfield, 2002; Pintrich and De Groot, 1990; Robbins et al., 2004). Moreover, motivation is a determinant of critical life decisions such as career, vocational, and course selection (Eccles, 2009); thus, educators are compelled to maximize students’ motivation as much as possible.

A considerable body of research in music education has focused on learner motivation, drawing on a variety of theoretical frameworks (for reviews, see Cogdill, 2015; Hallam, 2009; Oliveira et al., 2021; Sin et al., 2022). For example, self-determination theory (SDT) posits that the satisfaction of three basic psychological needs—autonomy, relatedness, and competence—facilitates a shift in learners’ motivation from extrinsic motivation, driven by external rewards, to intrinsic motivation, characterized by interest and enjoyment in the activity itself (e.g., Deci and Ryan, 2000; Evans, 2015). Based on SDT, Bonneville-Roussy and Evans (2024) found that autonomy support provided by music instructors was essential for enhancing intrinsic regulation among conservatoire students. Valenzuela et al. (2018), also drawing on SDT, investigated the effects of SDT-defined motivational constructs and psychological needs (perceived competence and autonomy) on flow experiences among conservatoire students. Their findings revealed that autonomous motivation (β = 0.21, *p* = 0.005) and perceived competence (β = 0.52, *p* < 0.001) had positive predictive effects, whereas controlled motivation had a negative effect. Research focusing on school-based music education has also been conducted. For instance, in a study of high school students, elective intentions to enroll in music courses were explained by both psychological needs—including competence—and value (Freer and Evans, 2018). Importantly, while SDT theoretically positions perceived competence as a causal factor that facilitates the emergence of autonomous or intrinsic motivation (rather than as a motivational variable in itself), empirical evidence has shown that perceived competence accounts for variance in a range of outcome variables even when autonomous or intrinsic regulation is statistically controlled (e.g., Krause et al., 2019; Valenzuela et al., 2018). Similar patterns have been observed in research grounded in theories other than SDT. For example, self-efficacy has been identified as a key predictor of musical performance quality (Ritchie and Williamon, 2012), and it is also significantly associated with self-esteem, grit, and learning and playing habits among amateur musicians (Harpaz and Vaizman, 2022). Taking these findings in music education into account, the present study adopts the expectancy-value theory (EVT), which explicitly conceptualizes “I can do this” -type beliefs—akin to perceived competence and self-efficacy—as central motivational component.

In recent years, research in music education based on EVT has been on the rise (Sin et al., 2022). The contemporary EVT frames motivation based on two primary components: “expectancy of success” and “subjective task value” (Eccles, 2009; Eccles and Wigfield, 2002; Wigfield and Eccles, 2000). The concept of the “expectancy of success” is defined as “students’ beliefs about how well they will do on upcoming tasks, either in the immediate or longer-term future” (e.g., Wigfield and Eccles, 2000), and can broadly be seen as the subjective response to the question “Can I do this?” (Bakhtiar and Hadwin, 2022). The notion of expectancy has been defined and interpreted in subtly different ways across various theories, leading to some confusion (jingle-jangle fallacies; e.g., Marsh et al., 2019). While similar to self-efficacy, expectancy of success in EVT is distinct in its definition, separating from perceived abilities or task difficulty. However, in empirical research, measures of expectancy are often found to constitute the same latent variable or are detected as highly correlated (Eccles and Wigfield, 2024; Marsh et al., 2019; Wigfield and Eccles, 2000), and thus, they are sometimes measured interchangeably in empirical studies based on EVT (e.g., Eccles and Wigfield, 2024; Nagengast et al., 2011; Trautwein et al., 2012). Considering this fact, the current study operationally defines the concept of expectancy as the perception or belief about an individual’s ability and the difficulty related to learning in music (Sin et al., 2022), referring to it as “expectancy belief.”

The concept of “subjective task value” comprises the individual’s interest or intrinsic value, importance or attainment value, usefulness or utility value, and cost as a negative value associated with the task (e.g., Anderman and Wolters, 2006; Eccles, 2009; Wigfield and Eccles, 2000), and can broadly be considered as the subjective response to the question “Is it worth doing?” (Bakhtiar and Hadwin, 2022). Proponents of EVT note that interest, or intrinsic value, is a component conceptually similar to intrinsic motivation in SDT (Wigfield and Eccles, 2000). Both expectancy belief and task value are associated with various learning behaviors, decision-making, and academic achievement; specifically, expectancy belief is known to strongly correlate with academic achievement or performance, while task value is strongly associated with choices of tasks, courses, or educational trajectories (e.g., Meece et al., 1990; Wigfield and Eccles, 2000).

The classic expectancy-value model posits a multiplicative relationship between expectancy beliefs and task value, assuming that comprehensive motivation, or the tendency to engage in behavior, is heightened when both are at elevated levels (e.g., Atkinson, 1957; Feather, 1982). Psychological research conducted after 2010 has succeeded in reviving this multiplicative relationship within Eccles et al.’s framework. Studies have adopted statistical models that assume a multiplicative relationship between the two motivational components for diverse outcome variables related to career choices in science-related fields and extracurricular science-related activities (Nagengast et al., 2011), academic achievement (Meyer et al., 2019; Trautwein et al., 2012), as well as undesirable behaviors such as procrastination and cheating tendencies (Lee et al., 2014).

Because the motivational components in EVT are domain-specific (e.g., Eccles and Wigfield, 2002, 2024; Eccles et al., 1993; Wigfield and Eccles, 2000), careful consideration must be given to how each concept should be differentiated and understood. Previous research has identified a significant disconnect between learning in school and engagement with music in everyday life (e.g., Boal-Palheiros and Hargreaves, 2001; Lamont et al., 2003; Ross, 1995, 1998), suggesting that motivation for formal school-based music learning and private, informal music listening should be measured separately. The terms “formal” and “informal” were originally described in the literature by L. Green. Green (2002) pointed out problems in the traditional “formal” approach to school-based music education and advocated for the incorporation of “informal” learning contexts into school-based music education, later providing practical pedagogical guidance (Green, 2008). In this context, the terms “formal” and “informal” refer to a contrast between learning within conventional music education settings and learning through unsupervised, trial-and-error processes, such as those found in the developmental pathways of popular musicians (Green, 2002, 2005, 2008). Folkestad (2006) reviewed the literature and organized the usage and definitions of these terms along four dimensions: (i) the situation, (ii) learning style, (iii) ownership, and (iv) intentionality. In the present study, motivation for school-based music education, which is often the direct target of intervention by school music teachers, is measured separately from motivation for private music listening. The former is operationally defined as motivation for formal music learning (MFM), meaning motivation that arises in the context of (i) school settings, (ii) learning based on textbooks and instructional materials, (iii) teacher-led instruction aligned with the national curriculum, and (iv) engagement with musical knowledge, performance skills, and cultural understanding. The latter is operationally defined as motivation for informal music engagement (MIM), referring to motivation that arises in the context of (i) out-of-school settings, (ii) self-directed music listening, (iii) fully self-determined behavior, and (iv) leisure-oriented engagement. Based on this distinction, the present study tests whether MFM can explain elementary school students’ music listening behaviors in everyday life—in other words, behaviors occurring in informal contexts—or whether MFM loses its explanatory power after controlling for MIM.

### 1.5 Current study

The primary aim of the present study is to examine whether MFM can explain students’ music listening behaviors in everyday life, as conceptualized by intention and attention. While MFM is the main independent variable of interest, it is expected to be positively correlated with MIM. Therefore, including only MFM in the model while ignoring MIM may lead to an overestimation of its effect size. To address this issue, both MFM and MIM are included as independent variables in the model, and the standardized partial regression coefficients are interpreted. Accordingly, the present study’s research question (RQ) can be stated as: “Can MFM explain variance in students’ everyday music listening behaviors (intention and attention) even after statistically controlling for MIM?” If the answer is “Yes,” then the partial regression coefficients from MFM to intention and attention are expected to be significantly positive. Answering this RQ may offer a provisional response to the simple yet important questions often posed in school-based music education: “Why should public education music teachers endeavor to enhance their students’ motivation?” and “What can elevating students’ motivation toward formal music learning change in their everyday behaviors?”

However, the absence of an expectancy belief scale adapted to the context of music education in Japan, despite the existence of a Japanese version of the task value scale (Kera and Nakaya, 2014), impedes the progress of research. Therefore, prior to achieving the main objective, we developed a psychological scale for expectancy belief for formal music learning and verified its validity (Analysis 1).

Subsequently, we addressed the primary RQ: “Can MFM explain students’ music listening behaviors in everyday life even after statistically controlling for MIM?” (Analysis 2). Here, music-listening behavior encompasses two aspects: the intention to access music in daily life (hereafter, intention) and attention to musical elements during everyday music listening (hereafter, attention). In this study, motivation is conceptualized as four factors arising from two components (expectancy belief and task value) across two contexts (MFM and MIM), forming a 2 × 2 factorial structure. The model estimated in this research is a regression model in which the main effects of the four factors (latent variables) and the two interaction terms (expectancy belief × task value for MFM and MIM, respectively) serve as the independent variables, with the two music-listening behaviors as the dependent variables.

## 2 Materials and methods

### 2.1 Creation of items for measuring expectancy belief for formal music learning

Considering the context of formal music education in Japan (MEXT, 2017), we developed items to measure expectancy belief for formal music learning through the following steps.

First, acknowledging that expectancy belief is task-dependent (e.g., Eccles and Wigfield, 2002; McPherson and O'Neill, 2010), we identified five specific domains under the global belief of “music learning” (instrumental performance, singing, composing, listening, and studying musical terminology) based on the Course of Study (MEXT, 2017), and decided to measure them separately.

Second, in addition to these domain-specific items, four items were prepared to represent the global expectancy belief of “music learning.” This procedure allowed us to examine whether a second-order factor, explaining the correlations among domain-specific expectancy beliefs, represents a domain-general expectancy belief.

Third, we maintained expressions that were comprehensible to elementary school students (from grades 4–6) and prepared items representing beliefs about abilities and perceived difficulty (Table 1).

**Table 1:** Items and descriptive statistics for EBFM.

Item		Mean	SD
G1	If I apply myself, I think I can do well in music classes	2.916	0.799
A1	I believe I can perform well in “playing an instrument” in music classes	2.646	0.881
B1	I believe I can perform well in “singing” in music classes	3.065	0.725
C1	I think I can do well in “creating music” activities in music classes	2.630	0.833
D1	I think I can do well in “music listening” activities in music classes	2.886	0.798
E1	I believe I can understand “musical terms” studied in music classes well	3.027	0.741
G2	I think I can achieve good grades in music classes	2.905	0.767
A2	I believe I can achieve good grades in “playing an instrument” in music classes	2.772	0.840
B2	I believe I can achieve good grades in “singing” in music classes	3.082	0.720
C2	I believe I can achieve good grades in “creating music” activities in music classes	2.770	0.770
D2	I think I can achieve good grades in “music listening” activities in music classes	3.024	0.786
E2	I believe I can understand “musical terms” studied in music classes and achieve good grades	3.008	0.750
G3	I think studying in music classes is easy	3.065	0.735
G4	I believe it is easy to get good grades in music classes	3.103	0.703
A3	I do not think that “playing an instrument” in music classes is easy	2.579	0.881
B3	I think “singing” in music classes is easy	3.486	0.581
C3	I think “creating music” activities in music classes are easy	2.970	0.714
D3	I think “music listening” activities in music classes are easy	2.949	0.718
E3	I believe that understanding “musical terms” studied in music classes is easy	3.118	0.667

A to E represent the principal learning contents in music classes as defined by the Japanese Course of Study (MEXT, 2017). G refers to music classes in general.

Fourth, we solicited opinions regarding the validity of the items from practicing elementary school teachers and made minor revisions accordingly.

### 2.2 Participants

The participants were 1,382 students (female: *n* = 640; male: *n* = 735; no response: *n* = 7) from grades 4 to 6, attending six public elementary schools in Japan on the day of the survey. Specifically, the sample included 4th-grade students (9 or 10 years old; *n* = 337), 5th-grade students (10 or 11 years old; *n* = 560), and 6th-grade students (11 or 12 years old; *n* = 485). These schools were located in regional cities and did not conduct entrance examinations for enrollment. Therefore, it was assumed that the students attending these schools had approximately average socioeconomic statuses (SESs) and academic abilities within the national context of Japan.

### 2.3 Measurements

The measurements of all items except for Expectancy Belief for Formal Music Learning (EBFM) are shown in Supplementary Table S1.

#### 2.3.1 MFM

##### 2.3.1.1 EBFM

The items relating to EBFM are shown in Table 1. Responses were solicited using a 4-point Likert scale (1: Does not apply, 2: Rather does not apply, 3: Somewhat applies, 4: Applies).

##### 2.3.1.2 Task value for formal music learning

A partially abbreviated version of the Japanese scale created by Kera and Nakaya (2014), which measures the positive task value excluding costs, was utilized. This scale comprises the interest value, attainment value, utility value in daily life, and utility value for entrance examinations and career formation. Considering that the majority of students in Japan advance to lower secondary school without entrance examinations, and music is rarely required for those exams even when they are present, the utility values related to entrance examinations and career formation were deemed implausible and thus omitted. The items used included statements such as “I enjoy studying in music classes” (interest value), “Understanding the content of music classes makes me feel that I can grow as a person” (attainment value), and “I believe that studying in music classes is beneficial in my everyday life” (utility value), among a total of 10 items. All items are listed in Supplementary Table S1. Responses were collected using a 4-point Likert scale, consistent with the scale used for EBFM.

#### 2.3.2 MIM

##### 2.3.2.1 Expectancy belief for informal music engagement

Based on the definition of expectancy belief, four items (e.g., “If I set my mind to it, I think I can easily listen to music regularly.”) were developed to suit everyday music listening. Responses were collected using a 4-point Likert scale, consistent with the scale used for EBFM.

##### 2.3.2.2 Task value for informal music engagement

The scale by Kera and Nakaya (2014) was modified to fit everyday music listening, which consists of 10 items (e.g., “I enjoy listening to music regularly,” see Supplementary Table S1). Responses were collected using a 4-point Likert scale, consistent with the scale used for EBFM.

#### 2.3.3 Music listening behavior in daily life

##### 2.3.3.1 Intention

The intention to listen to music in daily life was measured using three items (e.g., I regularly choose to listen to music on my own). Responses were collected using a 4-point Likert scale, consistent with the scale used for EBFM.

##### 2.3.3.2 Attention

The degree of attention paid to the eight elements constituting music, as defined by Japan’s Course of Study (MEXT, 2017), was measured during regular music listening. Items included statements such as “Usually pay attention to ‘rhythm’ when listening to music.” Responses were collected using a 4-point Likert scale, consistent with the scale used for EBFM.

#### 2.3.4 Variables used for validity assessment of EBFM

##### 2.3.4.1 Gender

Participants were asked to choose from three options: female, male, and “prefer not to say.” As will be discussed later, participants were informed that all item responses were optional; thus, those who chose “prefer not to say” and those who did not respond were treated similarly as “gender not disclosed.” Female was coded as 1, male as 0, and non-response or “prefer not to say” was treated as missing data and dummy-coded.

##### 2.3.4.2 SES

The short surrogate index for children’s SES using household possessions proposed by Ishii et al. (2019) was employed. This measure allows for a brief assessment of SES based on three household possessions (literary works, artworks, and dishwasher), which avoids ethical issues associated with SES measurement. Ishii et al. (2019) confirmed the validity of this SES measure by demonstrating significant positive correlations with subcomponents of SES (e.g., family income, parental education, and subjective class identification) and global SES scores, as well as high test–retest reliability among Japanese students. Participants were asked, “Do you have the following items in your household?” and responses were coded as 1 for “Yes” and 0 for “No.” The sum of the three items was used as the SES score. In general, SES is conceptualized not as a reflective measurement, such as those used in factor analysis, but as a formative measurement (e.g., Li and Wang, 2024). For formative indicators, high internal consistency is not required (Bollen and Lennox, 1991; Hwang et al., 2020). Therefore, in the present study, no reliability indices (e.g., α, ω) were calculated, and the total score of the three items was used as the SES index.

##### 2.3.4.3 Private music education

Students were asked whether they are currently receiving private music education (PME) outside of school (current PME participation; CPME) and whether they would like to receive private music education if it was available in the future (desire for PME if available; DPME). Each construct was measured using a single item: “I am currently taking music-related lessons outside of school classes (e.g., piano lessons)” for CPME, and “If possible, I would like to study music outside of school as well in the future” for DPME. Responses were coded as 1 for “Yes” and 0 for “No.”

### 2.4 Procedures and ethical considerations

This study was conducted in June, 2 months after the advancement period in Japanese educational institutions.

This research was carried out following approval received from the Ethics Committee of Akita University (No. 2023-004). The survey was conducted by the teaching staff of the cooperating schools. A procedural manual was prepared, and all participants took part in the survey under uniform conditions.

Prior to conducting the survey, permission was obtained from the administrative staff of participating schools. To ensure informed consent, information regarding the survey was sent in advance to the parents of the participating students via email or documents, with instructions to contact their affiliated schools if they chose to opt out of the study. Additionally, during questionnaire administration, teachers communicated the following both in writing and verbally: that participation was voluntary, that responses would be used solely for research purposes and would not affect grades, and that the data would be anonymized and subjected to statistical processing. The teachers conducting the survey were instructed, as per the procedural manual, not to view the students’ responses during survey completion. Because this survey was anonymous, the authors only had access to data that did not include personal information.

### 2.5 Statistical analysis

In this study, structural equation modeling (SEM) was employed to estimate the parameters. For Analysis 1, R (ver. 4.2.2; R Core Team, 2022) with the additional packages psych (ver. 2.2.9; Revelle, 2022) and lavaan (ver. 0.6.15; Rosseel, 2012) were used, while Mplus (ver. 8.4; Muthén and Muthén, 1998–2017) was used for Analysis 2. Robust maximum likelihood (MLR) was employed as the estimation method to account for the slight non-normality of the data. The missing values were estimated using the full information maximum likelihood method.

#### 2.5.1 Analysis 1

In Analysis 1, a factor analysis of the EBFM was initially conducted. Given that expectancy belief is considered a task-dependent concept, it is anticipated not to form a single factor, but rather to identify five latent variables unique to each context. We assessed this possibility using a Confirmatory Factor Analysis (CFA). Since we had *a priori* hypotheses regarding the factor structure of the EBFM, we did not conduct an exploratory factor analysis. Following common CFA conventions, we evaluated model fit using the following criteria: comparative fit index (CFI) > 0.95, Tucker–Lewis index (TLI) > 0.95, root mean square error of approximation (RMSEA) < 0.05, and standardized root mean square residual (SRMR) < 0.08. If these thresholds were not met, we considered the model to have failed to support the hypothesized measurement structure (e.g., Browne and Cudeck, 1992; Hu and Bentler, 1999).

Second, even if latent variables unique to the five contexts were identified, it is unlikely that they would form an orthogonal factor structure (with inter-factor correlations being zero). They are expected to have a highly correlated oblique structure. Taking into account that task value is measured in the global context of “music learning,” this study aimed to define a general expectancy belief factor for “music learning” by representing the correlations between the five factors with a second-order factor model. Notably, because a second-order factor model does not assume strict unidimensionality by nature, it is not appropriate to use Cronbach’s α as a reliability index (Revelle and Condon, 2019). Instead, it is recommended to report ω_h_ and ω_hc_ (Raykov et al., 2025). The coefficient ω_h_ is defined as the “proportion of the conventional scale score variance that is explained by the second-order factor” and is frequently reported in the literature (Raykov et al., 2025; Revelle and Condon, 2019; Zinbarg et al., 2006). The coefficient ω_hc_, defined as the “proportion of average observed scale component correlation that is attributable to, contributed, or explained by the second-order factor,” captures a different aspect of reliability from ω_h_, and it is therefore recommended to report both (Raykov et al., 2025). Accordingly, the present study focused on these two indices. However, users of the EBFM scale developed in this study may occasionally use unweighted summed scores of each subscale rather than weighted scoring. Although such models (i.e., those assuming essential tau-equivalence; e.g., McNeish and Wolf, 2020) rarely fit the data well, they are sometimes adopted for practical reasons. Therefore, Cronbach’s α was also calculated for each EBFM subscale to provide supplementary reliability estimates under the assumption of an essential tau-equivalent measurement model.

Third, there is no guarantee that the defined second-order factor represents a global expectancy belief in “music learning.” Therefore, this model was supplemented with four items measured against a general context of “music learning” to ensure they had sufficient factor loadings.

Fourth, previous research has shown that motivation toward music is higher in female students than in male students (McPherson and O'Neill, 2010) and tends to be greater with higher SES (Corenblum and Marshall, 1998). Furthermore, students receiving private music education (respondents who answered “Yes” to the CPME item) were presumed to have higher knowledge and skills related to music; consequently, a higher EBFM was anticipated. Based on the EVT, EBFM should predict decision-making related to task selection (e.g., Eccles and Wigfield, 2002; Wigfield and Eccles, 2000). Therefore, EBFM is expected to show a positive correlation with the DPME item.

#### 2.5.2 Analysis 2

First, ω coefficients were calculated as reliability estimates for each independent variable [Task value for formal music learning (TVFM), Expectancy belief for informal music engagement (EBIM), and Task value for informal music engagement (TVIM)] and each dependent variable (intention and attention) (Revelle and Condon, 2019). For EBIM, intention, and attention, McDonald’s ω was reported (McDonald, 1999). Since TVFM and TVIM were represented by second-order factors, ω_h_ and ω_hc_ were reported instead. The use of second-order factor models serves as an approach for deriving a higher-order abstraction of “task value,” while also avoiding multicollinearity that may arise from high correlations among the subcomponents of task value (e.g., Bagozzi and Yi, 2012; Chen et al., 2006; Koufteros et al., 2009).

Next, correlation coefficients among all variables included in the SEM were computed. Reporting correlation coefficients is recommended even when they are not directly related to the research questions, as they are useful for replicating SEM results and for use in future meta-analyses (McDonald and Ho, 2002; Schreiber et al., 2006; Schumacker and Lomax, 2016).

In Analysis 2, to address the main research question of this study, SEM was estimated with music listening behavior in daily life (intention, attention) as the dependent variable and MFM (EBFM, TVFM, EBFM × TVFM) and MIM (EBIM, TVIM, EBIM × TVIM) as the independent variables. The resolution of this research question was exploratory; therefore, no working hypotheses were formulated *a priori* regarding the expected signs of the estimated parameters. If a significant interaction was detected, we conducted a simple slopes analysis following conventional practice by substituting the moderator variable at ±1 SD into the model.

## 3 Results

### 3.1 Analysis 1: factor analysis for the EBFM scale

Factor analysis was performed on 15 items across five contexts (A–E). The initial eigenvalue inspection yielded a sequence of 7.18, 1.02, 0.91, 0.87, 0.64, 0.60. CFA assessed the fit for both a single-factor model and the hypothesized five-factor model. The chi-square test rejected the single-factor model (χ^2^ (90) = 918.617, *p* < 0.001), but did not reject the five-factor model (χ^2^ (80) = 92.722, *p* = 0.156). In addition, the five-factor model showed superior fit indices (CFI = 0.999, TLI = 0.998, RMSEA = 0.011, SRMR = 0.013) compared to the single-factor model (CFI = 0.910, TLI = 0.895, RMSEA = 0.084, SRMR = 0.047). All factor loadings for the five-factor model were high (λs = 0.605–0.872). To examine the possibility of cross-loadings within the five-factor model, we referred to the modification index (MI) and the standardized expected parameter change (SEPC). Only one item exceeded the MI threshold of 10.82 (F4 [listening factor] → C2 [composing item], MI = 12.745), but the SEPC value (0.170) was below the commonly accepted threshold (SEPC ≥ 0.2; Whittaker, 2011). Therefore, we concluded that there was no substantial cross-loading and retained all items as indicators of the EBFM scale. The Cronbach’s α coefficients for all five sub-scales (A–E) as well as the four items representing general music learning (G) were all at acceptable levels (A: α = 0.870, B: α = 0.739, C: α = 0.706, D: α = 0.760, E: α = 826, G: α = 0.916).

**Figure 1:**
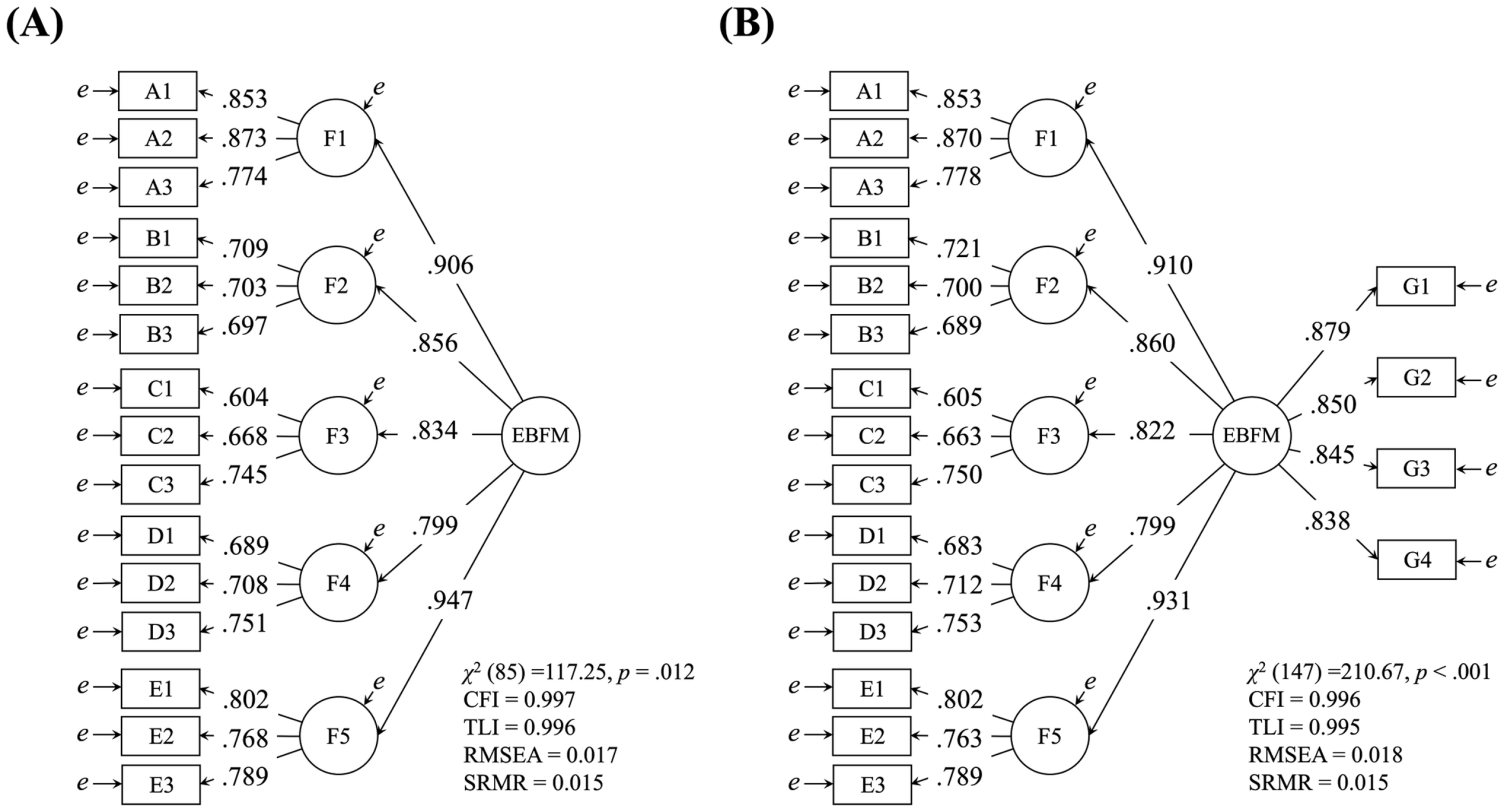
Results of the CFA for EBFM with standardized coefficients. Refer to Table 1 for details on each item. **(A)** A second-order model using items A to E, **(B)** A second-order model using items A to E and items G.

Subsequently, a model that introduced a second-order factor presumed to influence all five factors demonstrated good fit indices (Figure 1A). The reliability indices for the second-order factor model were also calculated, yielding ω_h_ = 0.883 and ω_hc_ = 0.958, indicating high reliability. Moreover, a model that hypothesized direct effects from the second-order factor to the four items measuring the general domain was also estimated to have good fit, and the factor loadings for the four items were sufficiently large (λs > 0.83; Figure 1B). Taken together, these results provide evidence for the validity and reliability of the EBFM scale and the factor structure developed in this study. Henceforth, this second-order factor is operationally defined as the “EBFM factor,” which was used for further analysis.

The model assuming covariances between the EBFM factor and gender, SES, CPME, and DPME was estimated using SEM. The model exhibited a good fit (CFI = 0.985, TLI = 0.982, RMSEA = 0.029, SRMR = 0.022), allowing for interpretation of the parameters. Significant positive correlations were observed, consistent with the hypotheses (Table 2). Consistent with the initial hypotheses, the EBFM factor showed significant positive correlations with two demographic variables (gender: *r* = 0.123, *p* < 0.001, SES: *r* = 0.174, *p* < 0.001) and PME (CPME: *r* = 0.470, *p* < 0.001, DPME: *r* = 0.429, *p* < 0.001). These results provide evidence for the convergent validity of the EBFM scale and the EBFM factor developed in this study.

**Table 2:** Correlation matrix between EBFM and the external variables (correlation parameters in CFA).

Variables		Mean	*SD*	1	2	3	4
1	EBFM	0.000	1.000				
2	Gender	0.465	0.498	0.123***			
3	SES	1.193	1.000	0.174***	−0.047		
4	CPME	0.263	0.440	0.470***	0.178***	0.177***	
5	DPME	0.579	0.493	0.429***	0.187***	0.081**	0.272***

EBFM, Expectancy Belief of Formal Music (latent variable); SES, Socioeconomic Status; CPME, Current Private Music Education; DPME, Desire for Private Music Education.

**p < 0.01, ***p < 0.001.

These findings supported the construct validity of the EBFM scale and the EBFM factor, prompting us to proceed to Analysis 2.

### 3.2 Analysis 2: SEM

Prior to conducting Analysis 2, a CFA was performed for the three variables: TVFM, EBIM, and TVIM (see Supplementary Table S2). The results indicated that all models had a satisfactory fit. Because there was a strong correlation between the three task values (interest value, attainment value, and utility value) for both TVFM and TVIM, it was posited that a higher-order latent variable could underlie these factors. Models including this higher-order factor showed a good fit, thereby supporting the use of a second-order factor reflecting a comprehensive cognition of task value for both the TVFM and TVIM indicators. The ω_h_ and ω_hc_ coefficients for both TVFM and TVIM were at satisfactory levels (Table 3). Therefore, in SEM, TVFM, and TVIM were modeled by a second-order factor representing “task value” and treated as independent variables.

**Table 3:** Reliability indices and correlation matrix for variables used in SEM.

Variables		Reliability			Correlation coefficients							
		ω	ω_h_	ω_hc_	1	2	3	4	5	6	7	8
1	Gender	–	–	–								
2	SES	–	–	–	−0.046							
3	Grade	–	–	–	0.011	0.007						
4	EBFM	–	0.883	0.958	0.123***	0.173***	0.050					
5	TVFM	–	0.799	0.910	0.278***	0.124***	0.025	0.778***				
6	EBIM	0.812	–	–	−0.018	0.109***	0.047	0.650***	0.458***			
7	TVIM	–	0.831	0.931	0.361***	0.153***	0.024	0.649***	0.684***	0.477***		
8	Intention	0.821	–	–	0.267***	0.125***	0.149***	0.631***	0.616***	0.575***	0.734***	
9	Attention	0.887	–	–	0.262***	0.172***	0.074**	0.767***	0.831***	0.570***	0.757***	0.665***

SES, Socioeconomic Status; EBFM, Expectancy Belief of Formal Music; TVFM, Task Value of Formal Music; EBIM, Expectancy Belief of Informal Music; TVIM, Task Value of Informal Music. EBFM, TVFM, EBIM, TVIM, intention, and attention are latent variables, whereas gender, SES, and grade are observed variables.

**p < 0.01, ***p < 0.001.

**Figure 2:**
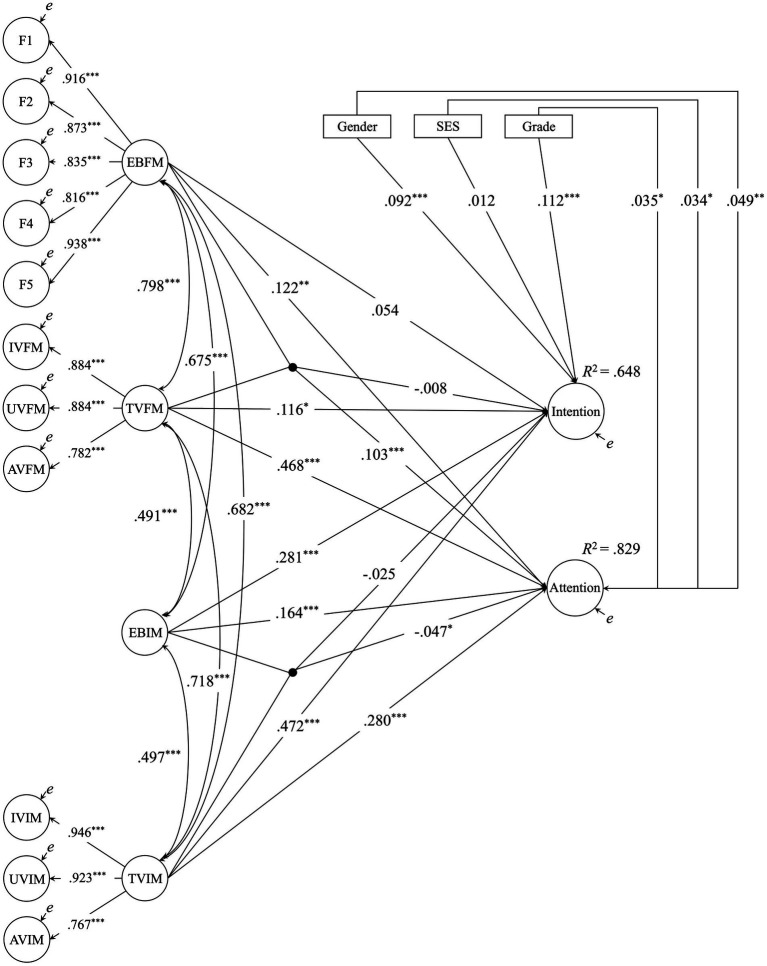
Estimated results of structural equation modeling (standardized coefficients). All standardized factor loadings for the items were of a sufficiently large size (all λs > 0.545, *ps* < 0.001). Covariances between the observed variables constituting each factor, factor loadings, and covariances between the control and explanatory variables are omitted for clarity. All measurement equations were based on a measurement model in which each item was explained by a single latent variable (first-order factor), and neither cross-loadings nor covariances among error terms were assumed. EBFM: expectancy belief for formal music learning (refer to Table 1 and Figure 1 for F1-F5), TVFM, task value for formal music learning; IVFM, interest value for formal music learning; UVFM, useful value for formal music learning; AVFM, attainment value for formal music learning; EBIM, expectancy belief for informal music engagement; TVIM, task value for informal music engagement; IVIM, interest value for informal music engagement; UVIM, useful value for informal music engagement; AVIM, attainment value for informal music engagement. The black dots represent latent interaction terms. *p < 0.05, **p < 0.01, ***p < 0.001.

Prior to conducting the SEM analysis, correlations among all independent variables (EBFM, TVFM, EBIM, TVIM), control variables (gender, SES, grade), and dependent variables (intention, attention) were examined. This model included gender, SES, and grade as observed variables in addition to the latent variables EBFM, TVFM, EBIM, TVIM, intention, and attention, and estimated covariances among all variables. That is, all unidirectional paths in Figure 2 were replaced with covariances, and no interaction terms were specified. The model demonstrated good fit (CFI = 0.989, TLI = 0.988, RMSEA = 0.015, SRMR = 0.022), and the estimated correlation coefficients were examined (Table 3). The dependent variables intention and attention showed significant positive correlations with all other variables in the model (*p*s < 0.01).

The estimated SEM results are shown in Figure 2. The control variables, grade, gender, and SES were centered at a mean of zero in the model. For models including latent interactions, fit indices were not available; therefore, we are unable to report them.

The results indicated that for intention, the main effects of TVFM, EBIM, and TVIM were significantly greater than zero, whereas the main effect of EBFM and the two interactions were not significant.

**Figure 3:**
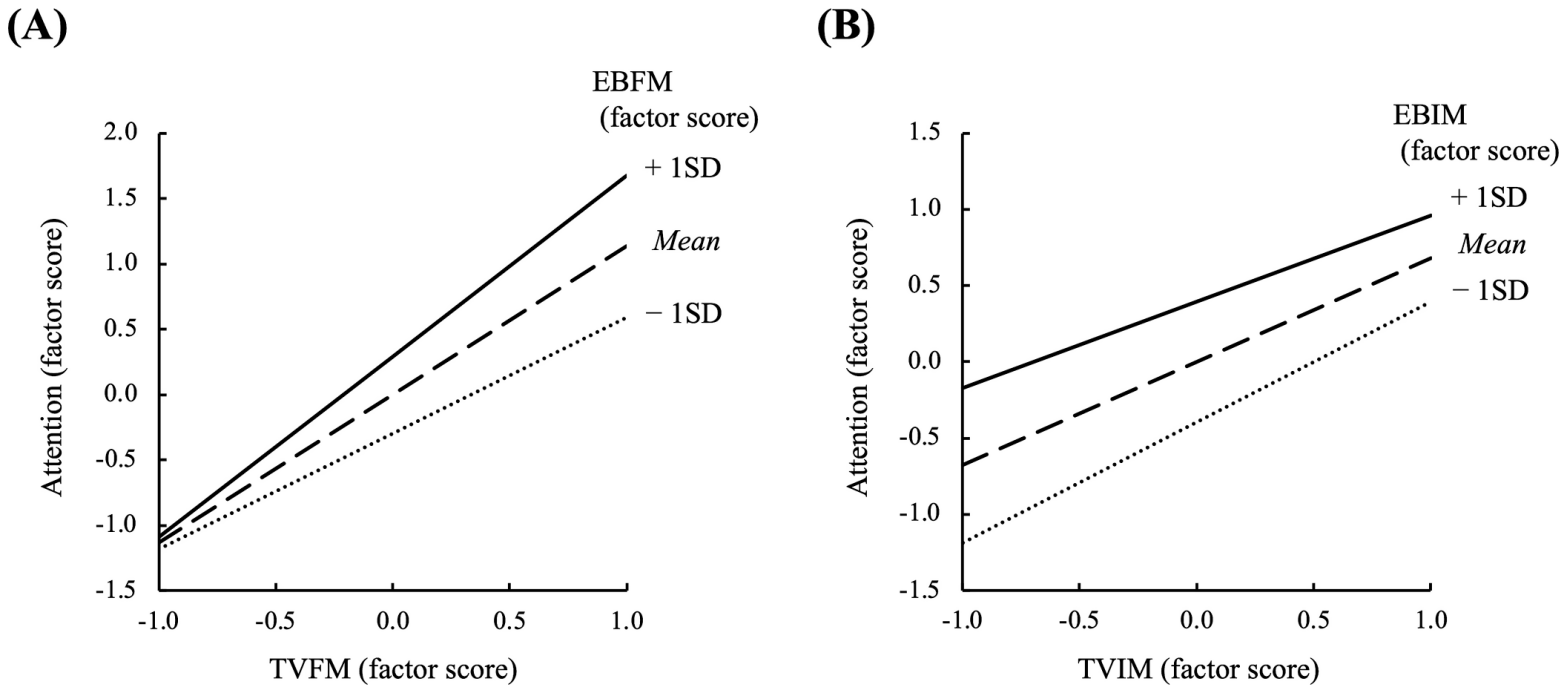
Visualization of simple slopes based on SEM estimates. **(A)** Interaction for MFM. **(B)** Interaction for MIM. Mean values have been inputted for all other explanatory variables (since factor means are defined as zero and control variables are centered, the mean value for all variables is zero). The latent mean of each dependent variable (i.e., the variable shown on the y-axis of each figure) was fixed at zero, and its residual variance (as distinct from the total variance) was constrained to 1.

For attention, the main effects of EBFM, TVFM, EBIM, and TVIM as well as the two interaction effects were significant. The interaction of MFM was positive, whereas that of MIM was negative. To visualize these interactions, Figure 3 depicts the graphs representing the simple slopes. In MFM, as TVFM increases, there is a tendency for the factor scores for attention to increase, with this effect being more pronounced in students with higher EBFM (Figure 3A). Conversely, in MIM, as TVIM increases, there is a tendency for the factor scores for attention to increase, with this effect being more pronounced in students with lower EBIM (Figure 3B).

## 4 Discussion

The objective of this study was to elucidate the effects of MFM within the school and MIM in daily life on music listening behaviors (intention, attention) among elementary school students. To achieve this goal, we first developed a measurement scale for EBFM that is suitable for the context of music education in Japan. The discussion proceeds in the order of the analyses conducted.

### 4.1 Nature and validity of the EBFM scale

The EBFM scale developed in this study has several distinct features. First, it reliably measures expectancy belief within five contexts of formal music education in Japan. This suggests that researchers and music teachers can measure expectancy belief in contexts specific to the learning unit targeted, which presents advantages for future music education research seeking to measure motivation unique to the learning unit. Second, there were moderate-to-strong positive correlations among expectancy beliefs unique to the five contexts, suggesting that these relationships originated from a higher-order expectancy belief toward general music learning. This implies that while expectancy belief is a task-specific construct (e.g., Eccles and Wigfield, 2002; McPherson and O'Neill, 2010), within the context of music education in elementary schools, it does not exhibit strong differentiation and retains a unidimensional structure. Third, the secondary factor of the EBFM scale, namely expectancy belief toward general formal music learning, was found to be higher for female students than for male students (*r* = 0.123), students with a higher SES (*r* = 0.174), and students who received private music education outside school (*r* = 0.470), supporting our initial hypothesis. Additionally, students with a higher secondary factor of EBFM were more likely to express a desire to pursue private music education outside school in the future (*r* = 0.429). These findings suggest that the item group developed possesses construct validity as a measure of expectancy belief toward formal music learning.

When evaluating the correlation between EBFM factors, gender, and SES against the conventional effect size benchmarks (Cohen, 1992), the level is considered “small.” The reasons for this modest level may be as follows. Concerning gender differences, a meta-analysis by Huang (2013) indicated that, generally, there are slight but higher gender differences in academic expectancy beliefs (self-efficacy) favoring male students (*g* = 0.08). However, these differences are not as pronounced in the early stages of development and are particularly significant in domains such as mathematics and the social sciences. Considering this point, the small yet significant superiority of female students found in EBFM in this study can be seen as reflective of the distinctive characteristics of music education. The small correlation between SES and EBFM could be considered for the following reasons. First, the SES scale used in this study was a simplified version comprising only three items (Ishii et al., 2019), which might have resulted in a lower reliability of SES measurement and subsequent attenuation of the correlation. Second, East Asian countries, including Japan, with a relatively homogeneous education system (e.g., Holloway et al., 2016), tend to show a weaker correlation between SES and academic achievement than Western countries (Kim, 2019). The weak correlation between EBFM and SES reported in this study may reflect a similar phenomenon. Although the results of this study do not clarify whether one of these reasons is true, or whether neither is correct, the fact that a significant correlation in the predicted direction was detected allows us to acknowledge the validity of the scale.

### 4.2 Effect of MFM and MIM on music listening behaviors in daily life

The primary RQ of this study was whether MFM could account for students’ music-listening behaviors in their daily lives, even when statistically controlling for MIM. The results of the SEM revealed significant positive partial regression coefficients for intention with TVIM (β = 0.472), EBIM (β = 0.281), and TVFM (β = 0.116). These findings suggest that the variables which predict the intention to engage in music listening in “daily life” relatively well are the perceived values toward music listening in daily life, and that the motivation toward formal music education in schools does not exert as much influence. Therefore, the inclination of Japanese elementary school students to listen to music in their daily lives is not closely related to their motivation toward music learning in schools, which might suggest that even if motivation within music lessons is enhanced, it may not significantly affect the “quantity” of music listening behavior in daily life.

For attention, significant main effects were observed for TVFM (β = 0.468), TVIM (β = 0.280), EBFM (β = 0.281), and EBIM (β = 0.122). The main effects of TVFM and EBFM on attention were also moderated by the typical interaction described as “expectancy × value” (e.g., Atkinson, 1957; Feather, 1982; Nagengast et al., 2011), with a coefficient β = 0.103. These results provide the answer “Yes” to the primary RQ of this study. Specifically, the two motivational factors for formal music learning imply a definition of the “quality” of music-listening behavior, such as “how” one listens to music. Students with higher expectancy beliefs toward music learning tend to listen to the components of music with attention, particularly when their recognition of the task value is elevated.

In contrast, the interaction between TVIM and EBIM was negative. Examining the simple slopes suggests that if either TVIM or EBIM is high, a certain degree of the attention factor score is discernible. This is distinct from the typical theoretical interaction, which suggests that a sufficient behavior arises only when both components are high. However, given the very small effect size of this interaction (β = −0.047), it may be prudent not to overinterpret the interaction effect. The interpretation and exploration of this phenomenon should be the subject of future studies.

Taken together, the results of the SEM provided a twofold answer to the primary RQ of this study. First, regarding intention—which represents the quantitative aspect of music listening—the effect of EBFM was not significant, and the effect of TVFM was minimal, whereas both EBIM and TVIM had significant positive effects. This suggests that the intention to access music in daily life is not well explained by motivational variables such as MFM, which school music teachers can relatively easily influence. This result aligns with a number of previous studies that have described a disconnect between school music education and students’ musical activities in everyday life (e.g., Boal-Palheiros and Hargreaves, 2001; Lamont et al., 2003; Ross, 1995, 1998). Although how students’ MIM (EBIM and TVIM) is formed remains unclear, given that the domain of measurement was “everyday life,” it is reasonable to assume that these variables are shaped primarily through experiences in everyday contexts. EVT provides a psychological framework for understanding how expectancy belief and task value—the two core components of motivation—are formed, and suggests that affective experiences, memories, and sociocultural beliefs are key determinants of task value (e.g., Eccles and Wigfield, 2002, 2024; Wigfield and Eccles, 2000). Accordingly, if music teachers aim to foster students’ intention to engage in everyday music listening, it may be effective to enhance MIM by encouraging students to have emotionally positive experiences and to recognize personal usefulness in contexts that closely resemble their everyday lives. This approach is closely aligned with prior pedagogical frameworks that incorporate informal music-learning contexts into formal music education (e.g., Green, 2002, 2005, 2008) or utilize music that is personally relevant to students’ lives (Todd and Mishra, 2013), and the present findings can be interpreted as empirical support for these educational strategies.

Second, with regard to attention, which represents the qualitative aspect of music listening, the finding indicated that the expectancy × value model of EBFM and TVFM (e.g., Atkinson, 1957; Nagengast et al., 2011) also function in the domain of music listening. This suggests that there is not a complete disconnection between school music education and students’ everyday music listening. That is, motivational variables promoted in formal music education can foster students’ use of attentive and analytical listening strategies toward musical elements in their daily lives. The use of such strategies has been associated not only with the facilitation of semantic processing of music (Daltrozzo and Schön, 2009; Koelsch et al., 2004; Painter and Koelsch, 2011), but also with emotion and stress regulation (Leipold and Loepthien, 2015), flow experiences induced by complex music (Ruth et al., 2017), and the understanding of music’s formal structures (Campbell, 2005). Thus, enhancing students’ motivation toward formal music education in school may promote meaningful engagement with music in their daily lives—even without directly intervening in their motivation for everyday music listening. This may be encouraging for music teachers, who are constrained by the national curriculum (in the case of Japan, MEXT, 2017) and may not be permitted to freely incorporate informal contexts into classroom instruction based on personal discretion.

This study answers the question, “Why should public education music teachers endeavor to enhance their students’ motivation?” The existing gap between formal music learning and students’ everyday music listening practices has increasingly complicated this inquiry, essentially leading to the question “What can elevating students’ motivation toward formal music learning change in their everyday behaviors?” The results of this study indicate that motivation in formal music education can promote attentive music-listening behaviors in daily life. The literature shows that attentive music listening enables the extraction of meaning from music (Daltrozzo and Schön, 2009; Painter and Koelsch, 2011), and offers a potential foundation for deep understanding and creativity in music as an art form (e.g., Campbell, 2005; Gordon, 1989; Peterson, 2006). These findings suggest that the state of motivation toward music learning in schools can influence how students experience, interpret, and create music in their daily lives.

### 4.3 Limitations

In the present study, music listening behavior in everyday life was operationalized by distinguishing between its quantitative aspect (intention) and qualitative aspect (attention), both of which served as dependent variables. However, music listening behavior serves diverse functions and purposes, and thus the motivational effects identified in this study may capture only a limited portion of the phenomenon. In particular, for adolescents, music listening is a culturally embedded practice that fulfills multiple roles (Miranda, 2012), including esthetic appreciation for music and the development of musical tastes (Hargreaves et al., 2006; Mulder et al., 2010), the construction of cultural identity and self-concept (Kistler et al., 2010), and the differentiation between in-group and out-group membership through musical preferences (Hargreaves et al., 2006). Moreover, the present study did not address the emotional aspects of music listening that have been investigated in previous research, such as emotional experiences, emotional expression, and emotion regulation (e.g., Garrido et al., 2022; Juslin et al., 2008; Leipold and Loepthien, 2021). Therefore, future research should examine how students’ motivation for school-based music education (i.e., MFM) relates to various dimensions of music listening behavior outside of school.

In this study, we distinguished two distinct contexts—formal (MFM) and informal (MIM)—and measured motivational components based on EVT within each context. However, in the fields of music psychology and music education, the terms “formal” and “informal” are often used as convenient labels rather than strictly dichotomous categories (Folkestad, 2006; Smart and Green, 2017). Therefore, future research may benefit from refining the mathematical modeling of MFM and MIM, as well as examining their interaction effects or causal relationships.

Owing to the observational nature of this study, which relies on a single-time-point measurement, it cannot address the causal relationship between motivation and music-listening behaviors. Although this study implicitly assumed that motivation (MFM, MIM) influences music listening behavior, several previous studies have suggested that music listening can in turn enhance one’s sense of agency—a concept closely related to self-efficacy—as well as motivation (e.g., Saarikallio et al., 2020; Vigl et al., 2023). Future research should aim to identify strict causal relationships through longitudinal studies that analyze Granger causality between the two variables or examine the effects of interventions on motivation and everyday music-listening behaviors. Additionally, because the participants of this study were limited to Japanese elementary school students, the generalizability of the findings needs to be considered carefully. Results from comparative studies involving other developmental stages or cultural regions are required to attain universal conclusions.

## Data Availability

The datasets presented in this article are not readily available because the administrators of two out of the six schools did not consent to public sharing of the data. Requests to access the datasets should be directed to the corresponding author. Requests to access the datasets should be directed to Yuki Harada, yukiharada@ed.akita-u.ac.jp.
