# Supplementary material for: Beyond school gates: the role of motivation in music learning on elementary school students’ daily music listening behaviors
Source: Front Psychol. 2025 Jul 16;16:1441572. doi: 10.3389/fpsyg.2025.1441572 (PMC12308851; doi:10.3389/fpsyg.2025.1441572)
Supplement: Supplementary file 1 [file Table_1.docx]

Supplementary Material

**Supplementary Table 1. List of Questionnaire Items**
