# Supplementary material for: Beyond school gates: the role of motivation in music learning on elementary school students’ daily music listening behaviors
Source: Front Psychol. 2025 Jul 16;16:1441572. doi: 10.3389/fpsyg.2025.1441572 (PMC12308851; doi:10.3389/fpsyg.2025.1441572)
Supplement: Supplementary file 2 [file Table_2.docx]

Supplementary Material

**Supplementary Table 2. Model Fit Indices for Confirmatory Factor Analysis**

**
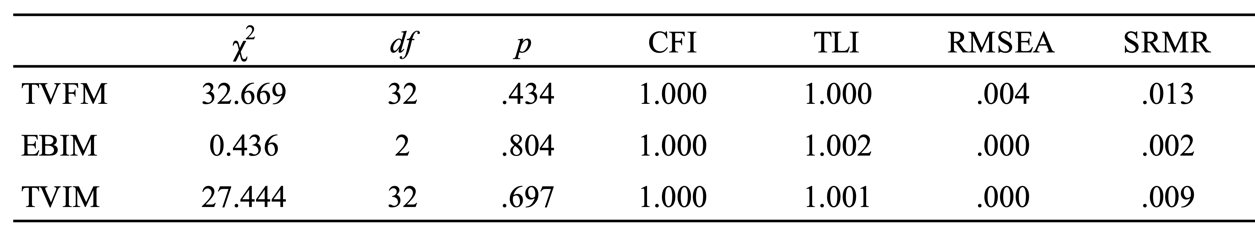
**

**Note:** *The TVFM and TVIM models are three-factor models allowing correlations among factors, whereas the EBIM model is a single-factor model.*
